# Novel variants and phenotypic heterogeneity in a cohort of 11 Chinese children with Wiedemann-Steiner syndrome

**DOI:** 10.3389/fgene.2023.1085210

**Published:** 2023-03-21

**Authors:** Yunting Lin, Xiaohong Chen, Bobo Xie, Zhihong Guan, Xiaodan Chen, Xiuzhen Li, Peng Yi, Rong Du, Huifen Mei, Li Liu, Wen Zhang, Chunhua Zeng

**Affiliations:** ^1^ Department of Genetics and Endocrinology, Guangzhou Women and Children’s Medical Center, Guangzhou Medical University, Guangdong Provincial Clinical Research Center for Child Health, Guangzhou, China; ^2^ Department of Endocrinology and Metabolism, Wuhan Children’s Hospital, Tongji Medical College, Huazhong University of Science and Technology, Wuhan, China; ^3^ Center for Medical Genetics and Genomics, The Second Affiliated Hospital of Guangxi Medical University, Nanning, China; ^4^ The Guangxi Health Commission Key Laboratory of Medical Genetics and Genomics, The Second Affiliated Hospital of Guangxi Medical University, Nanning, China

**Keywords:** Wiedemann-Steiner syndrome, *KMT2A* gene, clinical characteristics, genetic spectrum, therapeutic effect, Chinese

## Abstract

**Objective:** Wiedemann-Steiner syndrome (WSS) is a rare autosomal dominant disorder caused by deleterious heterozygous variants of the *KMT2A* gene. This study aims to describe the phenotypic and genotypic features of Chinese WSS patients, and assess therapeutic effects of recombinant human growth hormone (rhGH).

**Methods:** Eleven Chinese children with WSS were enrolled in our cohort. Their clinical, imaging, biochemical and molecular findings were analyzed retrospectively. Moreover, the phenotypic features of 41 previously reported Chinese WSS patients were reviewed and included in our analysis.

**Results:** In our cohort, the 11 WSS patients presented with classic clinical manifestations, but with different frequencies. The most common clinical features were short stature (90.9%) and developmental delay (90.9%), followed by intellectual disability (72.7%). The most frequent imaging features were patent ductus arteriosus (57.1%) and patent foramen ovale (42.9%) in cardiovascular system, and abnormal corpus callosum (50.0%) in the brain. In the set comprising 52 Chinese WSS patients, the most common clinical and imaging manifestations were developmental delay (84.6%), intellectual disability (84.6%), short stature (80.8%) and delayed bone age (68.0%), respectively. Eleven different variants, including three known and eight novel variants, of the *KMT2A* gene were identified in our 11 WSS patients without a hotspot variant. Two patients were treated with rhGH and yielded satisfactory height gains, but one developed acceleration of bone age.

**Conclusion:** Our study adds 11 new patients with WSS, reveals different clinical characteristics in Chinese WSS patients, and extends the mutational spectrum of the *KMT2A* gene. Our study also shares the therapeutic effects of rhGH in two WSS patients without GH deficiency.

## 1 Introduction

Wiedemann-Steiner syndrome (WSS) is a rare autosomal dominant disorder caused by deleterious heterozygous variants of the *KMT2A* gene located on chromosome 11q23.3. The *KMT2A* gene, also known as the *MLL* gene, is comprised of 36 exons with a range of 16.6 Kb that encodes a histone lysine methyltransferase of 3,969 amino acids (Jones et al., 2012). The KMT2A protein comprises a number of functional domains: three DNA binding AT hooks, a CXXC zinc finger domain, four PHD zinc finger domains, a BD domain, a FYRN domain, a TAD motif, a FYRC domain, a WIN motif, and a SET domain (Jones et al., 2012; Aggarwal et al., 2017; Baer et al., 2018). To date, 349 different *KMT2A* variants, including 273 disease-causing and 76 possible disease-causing variants, have been documented in the Human Gene Mutation Database (HGMD, Professional 2022.4, https://www.hgmd.cf.ac.uk/) to cause WSS or other related disorders or phenotypes.

As the *KMT2A* gene regulates the expression of multiple *HOX* and *WNT* genes and plays a critical role in early development and hematopoiesis, WSS patients present complex and variable phenotypes involving multiple systems (Aggarwal et al., 2017; Baer et al., 2018; Li et al., 2018). The classic features of WSS include developmental delay, intellectual disability, short stature, facial dysmorphism, and hypertrichosis cubiti (hairy elbows). Other additional phenotypes of feeding difficulties, epilepsy, ocular abnormalities, congenital heart disease, musculoskeletal problems, genitourinary anomalies, endocrinologic issues, immunologic dysfunction, behavioral differences, and abnormal brain magnetic resonance imaging (MRI) also have been described in WSS (Miyake et al., 2016; Baer et al., 2018; Li et al., 2018; Sheppard et al., 2021; Castiglioni et al., 2022; Durand et al., 2022).

However, the features of WSS are shared by a range of disorders, including Coffin-Siris syndrome, Nicolaides-Baraitser syndrome, Cornelia de Lange syndrome, Rubinstein-Taybi syndrome, Kabuki syndrome, Bohring-Opitz syndrome, Suleiman-El-Hattab syndrome, and Blepharophimosis-ptosis-epicanthus inversus syndrome (Bramswig et al., 2015; Miyake et al., 2016; Baer et al., 2018; Li et al., 2018; Negri et al., 2019; Jinxiu et al., 2020; Demir et al., 2021; Di Fede et al., 2021; Sheppard et al., 2021; Castiglioni et al., 2022; Riedhammer et al., 2022). The overlapping clinical signs may complicate the diagnosis of a patient. Next-generation sequencing (NGS), including whole genome sequencing (WGS), whole exome sequencing (WES) and target region sequencing (TRS), may contribute to the precise diagnosis of WSS (Bramswig et al., 2015; Negri et al., 2019; Jinxiu et al., 2020; Demir et al., 2021; Di Fede et al., 2021).

Due to highly clinical heterogeneity and phenotypic similarity with other disorders, WSS is far away from well-known. The prevalence of WSS is estimated to be less than 1/1,000,000 (Luo et al., 2021). To date, less than 400 WSS patients have been reported in the worldwide, and some affected adults are not yet recognized until their child’s diagnosis (Sheppard et al., 2021). Thus, it is probable that a number of WSS cases are underdiagnosed.

In this study, we analyzed the clinical and genetic findings of 11 Chinese children with WSS retrospectively, and reviewed the clinical presentations of 41 previously reported Chinese WSS patients, in order to better describe phenotypic characteristics and mutational spectrum of Chinese WSS patients. We also evaluated the therapeutic effects of recombinant human growth hormone (rhGH) in two patients.

## 2 Materials and methods

### 2.1 Patients

Eleven WSS children from 11 unrelated families were enrolled in our cohort. Among them, eight patients (P1-P8) were enrolled in Guangzhou Women and Children’s Medical Center, one patient (P9) was enrolled in the Second Affiliated Hospital of Guangxi Medical University, and two patients (P10 and P11) were enrolled in Wuhan Children’s Hospital. All subjects are Chinese. P9 is of Zhuang ethnicity, while the other 10 patients are of Han ethnicity. All parents are asymptomatic and non-consanguineous.

### 2.2 Clinical information

The clinical information was collected and evaluated by clinicians. The physical examinations were performed by physicians, skeletal X-ray and brain MRI or computed tomography (CT) were performed by radiologists, ultrasonography was performed by sonologists, electrocardiogram (ECG) was performed by cardiologists, and electroencephalogram (EEG) was performed by neurologists. Biochemical parameters of blood samples were detected in hospital’s clinical laboratory center, and serum hormones were detected in hospital’s endocrinology and metabolism laboratory.

### 2.3 WES

Eleven patients were subjected to WES with a proband-parents trio or proband-only strategy. As described previously (Lin et al., 2020), genomic DNA (gDNA) was extracted from peripheral blood samples using DNeasy Blood and Tissue Kit (QIAGEN, Hilden, Germany). The workflow of WES was strictly according to the manufacturers’ protocol. gDNA was randomly interrupted to an average size of 180–280 bp by Covaris S220 ultrasonicator (Covaris, Woburn, United States). The fragmented products were end repaired and phosphorylated, followed by A-tailing and ligation at the 3’ ends with paired-end adaptors (Illumina, San Diego, United States). Subsequently, the prepared DNA library was purified using Agencourt AMPure SPRI beads (Beckman Coulter, Brea, United States) and detected by Agilent 2,100 Bioanalyzer and real-time PCR (Agilent, Santa Clara, United States). At last, the exome sequences were enriched from the qualified library using Agilent SureSelect Human All Exon V6 kit (Agilent, Santa Clara, United States) and sequenced on Illumina Novaseq 6,000 platform (Illumina, San Diego, United States) for paired-end 150 bp reads. The acquired data were processed upon an established analysis pipeline for variant calling and functional annotation to identify the disease-causing variants.

### 2.4 Molecular analysis of the *KMT2A* gene

To validate the candidate causative mutational site identified by WES, the classic Sanger sequencing was carried out using primers targeting the specific exons of the *KMT2A* gene (NG_027813.1, NM_001197104.2). The exon sequences together with adjoining intron boundaries were amplified by polymerase chain reaction (PCR) and sequenced using an ABI 3730xl DNA Analyzer (Applied Biosystems, Foster City, United States). The sequencing chromatograms were read by Chromas software (Technelysium, South Brisbane, Australia), while the exported sequences were aligned with the reference sequence using DNAMAN software (Lynnon Corporation, Vaudreuil, Canada). In addition, for gross deletions covering several exons of the *KMT2A* gene, a quantitative PCR (qPCR) of gDNA was conducted using a BIO-RAD CFX96 Real-Time System (BIO-RAD, Hercules, United States).

The dbSNP database (https://www.ncbi.nlm.nih.gov/snp/) was employed to exclude the polymorphic alleles, while HGMD and ClinVar (https://www.ncbi.nlm.nih.gov/clinvar/) databases were engaged for the confirmation of known pathogenic variants. For novel variants, the pathogenicity was evaluated according to the ACMG guidelines (Richards et al., 2015).

### 2.5 Treatment and follow-up

Five patients (P1, P4, P5, P8 and P10) in our cohort had clinic follow-ups, while three of them (P4, P5 and P10) kept regular clinic visits. Growth hormone (GH) provocation test and serum insulin-like growth factor (IGF-1) were assessed in these three patients.

Two patients (P4 and P10) were treated with rhGH (Jintropin, GeneScience Pharmaceuticals, Changchun, China). Clinical follow-up of P4 was performed with an interval of 1 month in the first 3 months after treatment and subsequently with an interval of 3 months, and her height, weight and serum IGF-1 were measured at every visit. P10 visited clinic with an interval of 3–6 months, but had a bad compliance as only her serum IGF-1 was monitored at every visit whereas her height and weight were measured irregularly.

### 2.6 Statistical analysis

SPSS Statistics 17.0 software (SPSS, Chicago, United States) was used to calculate means and standard deviations. Prism 6 GraphPad (GraphPad, San Diego, United States) was used to draw curve and line charts.

## 3 Results

### 3.1 Clinical characteristics

Among 11 WSS children in our cohort, eight were males and three were females, with a male-to-female ratio of 2.7:1. Most of them (7/10, 70.0%) presented prenatal growth retardation with a birth length of 45.5 ± 3.3 cm and a birth weight of 2.60 ± 0.47 kg at full-term gestation, and all of them showed postnatal growth retardation with a height standard deviation score (SDS) of −3.6 ± 1.3 and a weight SDS of −3.7 ± 1.5 at the first visit (Li et al., 2009) (Supplementary Table S1).

As shown in Supplementary Table S1, the clinical features of 11 WSS patients were highly complex and variable. The most frequent clinical features were short stature (10/11, 90.9%) and developmental delay (10/11, 90.9%), followed by intellectual disability (8/11, 72.7%). Other common clinical features presented in more than half of WSS patients included low-set ears, slim and muscular build, thick eyebrows, long eyelashes, and hypertrichosis of the back.

Skeletal X-ray, brain MRI or CT, abdominal and urinary ultrasonography, cardiac ultrasonography, ECG, and EEG were conducted in six, ten, five, seven, seven, and four patients, respectively. The most frequent imaging features were patent ductus arteriosus (4/7, 57.1%) and patent foramen ovale (3/7, 42.9%) in cardiovascular system, and abnormal corpus callosum (5/10, 50.0%) in the brain (Supplementary Table S1).

All the 11 patients showed normal liver, renal and thyroid functions, and no obvious signs of metabolic acidosis were revealed.

In addition, to fully investigate the phenotypic spectrum of Chinese WSS patients, 41 of 43 previously reported Chinese WSS patients with available identify information were reviewed and included in our analysis [(Sun et al., 2017; Gao et al., 2018; Li et al., 2018; Chen et al., 2019; Dai et al., 2019; Shangguan et al., 2019; Jinxiu et al., 2020; Wang et al., 2021a; Wang et al., 2021b; Feng et al., 2021; Lee et al., 2021; Luo et al., 2021; Sheppard et al., 2021; Xue et al., 2021; Chen et al., 2022; Wu et al., 2022; Yu et al., 2022), and other 4 literatures in Chinese], which increases the total sample size to 52.

Phenotypic heterogeneity was also shown in this set comprising 52 Chinese WSS patients. The male-to-female ratio was 1.3:1. Over half of them (23/45, 51.1%) and most of them (48/52, 92.3%) had prenatal and postnatal growth retardation, respectively. The most frequent clinical features were developmental delay (44/52, 84.6%), intellectual disability (44/52, 84.6%), and short stature (42/52, 80.8%). Other common clinical features presented in more than half of patients included hypertelorism, flat nasal bridge, thick hairs, long eyelashes, hypertrichosis of the back, hypertrichosis of lower limbs, and low hairline. The most frequent imaging feature was delayed bone age (17/25, 68.0%) (Supplementary Table S1).

### 3.2 Mutational spectrum

Eleven different variants, including three known and eight novel variants, of the *KMT2A* gene were identified in 11 WSS patients. All of them were heterozygous with a *de novo* pattern. According to the ACMG guidelines, all eleven *KMT2A* variants (six truncating, three missense, one splicing and one gross deletion) were classified as pathogenic or likely pathogenic (Table 1).

**TABLE 1:** Deleterious *KMT2A* variants identified in 11 Chinese WSS patients.

Patient	Exon	Variant	Allele	Inheritance	Reported previously?	ACMG criteria	
						Evidence	Classification
P1	3	c.2641G>T(p.Glu881*)	Het	*De novo*	Novel	PVS1 + PS2 + PM2 _Supporting + PP3 + PP4	Pathogenic
P2	5	c.3509G>T(p.Cys1170Phe)	Het	*De novo*	Novel	PS2 + PM2 _Supporting + PP3 + PP4	Likely pathogenic
P3	19	c.5431C>T(p.Arg 1811*)	Het	*De novo*	Ref (Fontana et al., 2020)	PVS1 + PS2 + PM2 _Supporting + PP3 + PP4	Pathogenic
P4	5	c.3503G>T(p.Gly1168Val)	Het	*De novo*	Novel	PS2+PM2 _Supporting + PP3 + PP4	Likely pathogenic
P5	12	c.4504C>T(p.Arg1502*)	Het	*De novo*	Ref (Sheppard et al., 2021)	PVS1 + PS2 + PM2 _Supporting + PP3 + PP4	Pathogenic
P6	32	c.11206C>T(p.Gln3736*)	Het	*De novo*	Novel	PVS1 + PS2 + PM2 _Supporting + PP3 + PP4	Pathogenic
P7	8	c.4038dupA(p.Val1347Serfs*24)	Het	*De novo*	Novel	PVS1 + PS2 + PM2 _Supporting + PP3 + PP4	Pathogenic
P8	5	c.3463T>A(p.Cys1155Ser)	Het	*De novo*	Novel	PS2+PM2 _Supporting + PP3 + PP4	Likely pathogenic
P9	5	c.3409A>T(p.Arg1137*)	Het	*De novo*	Novel	PVS1 + PS2 + PM2 _Supporting + PP3 + PP4	Pathogenic
P10	6	c.3570-1G>C	Het	*De novo*	Novel	PVS1 + PS2 + PM2 _Supporting + PP3 + PP4	Pathogenic
P11	2-10	ex.2-10 del	Het	*De novo*	Ref (Retterer et al., 2016)	PVS1 + PS2 + PM2 _Supporting + PP3 + PP4	Pathogenic

Het, Heterozygous.

**FIGURE 1:**
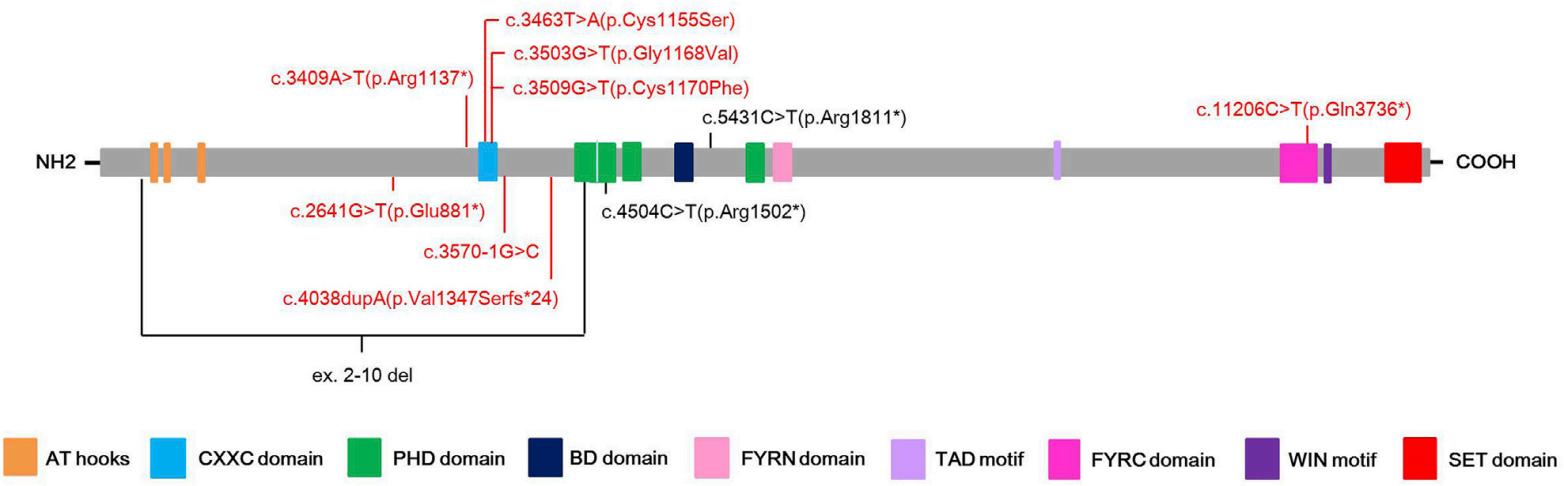
Schematic presentation of the KMT2A protein with domain structure and the localization of the eleven different variants found in this study. Red color highlights the novel variants, while black color stands for known pathogenic variants.

Among eleven identified *KMT2A* variants, six (6/11, 54.5%) were occurred in functional domains of the KMT2A protein: c.3463T>A(p.Cys1155Ser), c.3503G>T(p.Gly1168Val) and c.3509G>T(p.Cys1170Phe) missense variants were located at the CXXC domain; c.4504C>T(p.Arg1502*) nonsense variant was located at the PHD domain; c.11206C>T(p.Gln3736*) nonsense variant was located at the FYRC domain; while ex.2-10 del covered the AT hooks, CXXC domain and a part of PHD domain. Of the other five variants, four loss-of-function (LoF) variants, including c.2641G>T(p.Glu881*) and c.3409A>T(p.Arg1137*) nonsense variants, c.3570-1G>C splicing variant, and c.4038dupA(p.Val1347Serfs*24) frameshift variant, were distributed near by the CXXC domain, while c.5431C>T(p.Arg1811*) nonsense variant was close to the BD domain (Figure 1).

### 3.3 Treatment and follow-up

In our cohort, five patients (5/11, 45.5%) had clinic follow-ups ranging from 5 months to 2.3 years. Among them, P4, P5 and P10 had regular visits. They all showed short stature below −2 height SDS at the first visit and underwent GH provocation test to check for GH deficiency (GHD) (Supplementary Table S1). The GH peak level and IGF-1 level of P5 were 5.65 ng/mL and 36.6 ng/mL (reference range of IGF-1: 50–286 ng/mL) respectively, indicating GHD, whereas no evidence supported GHD in P4 and P10. Consistently, not P4 and P10 but P5 presented with delayed bone age at examination. However, only P4 and P10 received rhGH treatment based on low height gain and the parents’ permission.

**FIGURE 2:**
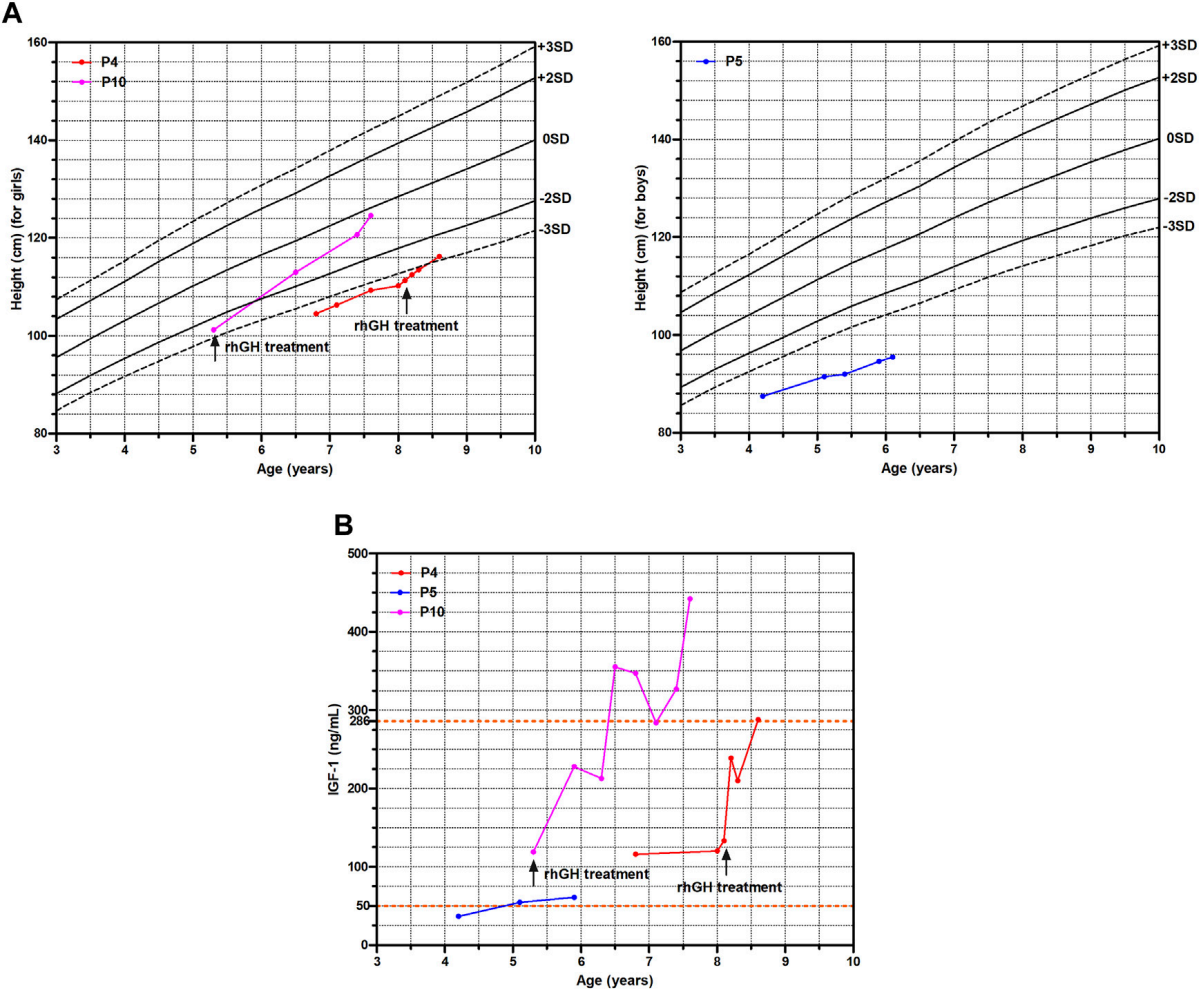
The growth curve and IGF-1 levels of three WSS children. **(A)** Growth curve. **(B)** IGF-1 levels.

P4 and P10 started rhGH treatment at 8.1 years old and 5.3 years old with a dose of 0.12 IU/kg/day and 0.14  IU/kg/day respectively. Comparing with P5 without a medical intervention, the growth trajectories of P4 and P10 improved significantly with a catch-up growth (Figure 2A), and their serum IGF-1 levels also markedly increased (Figure 2B). The height SDS of P10 increased from −2.6 to −0.3 after rhGH treatment of 2.3 years as well as P4 from −3.4 to −2.8 after rhGH treatment of 6 months, whereas P5 remained of a short stature with the height SDS from −4.6 to −5.0 during 1.9 years follow-up (Figure 2A; Supplementary Table S1).

**FIGURE 3:**
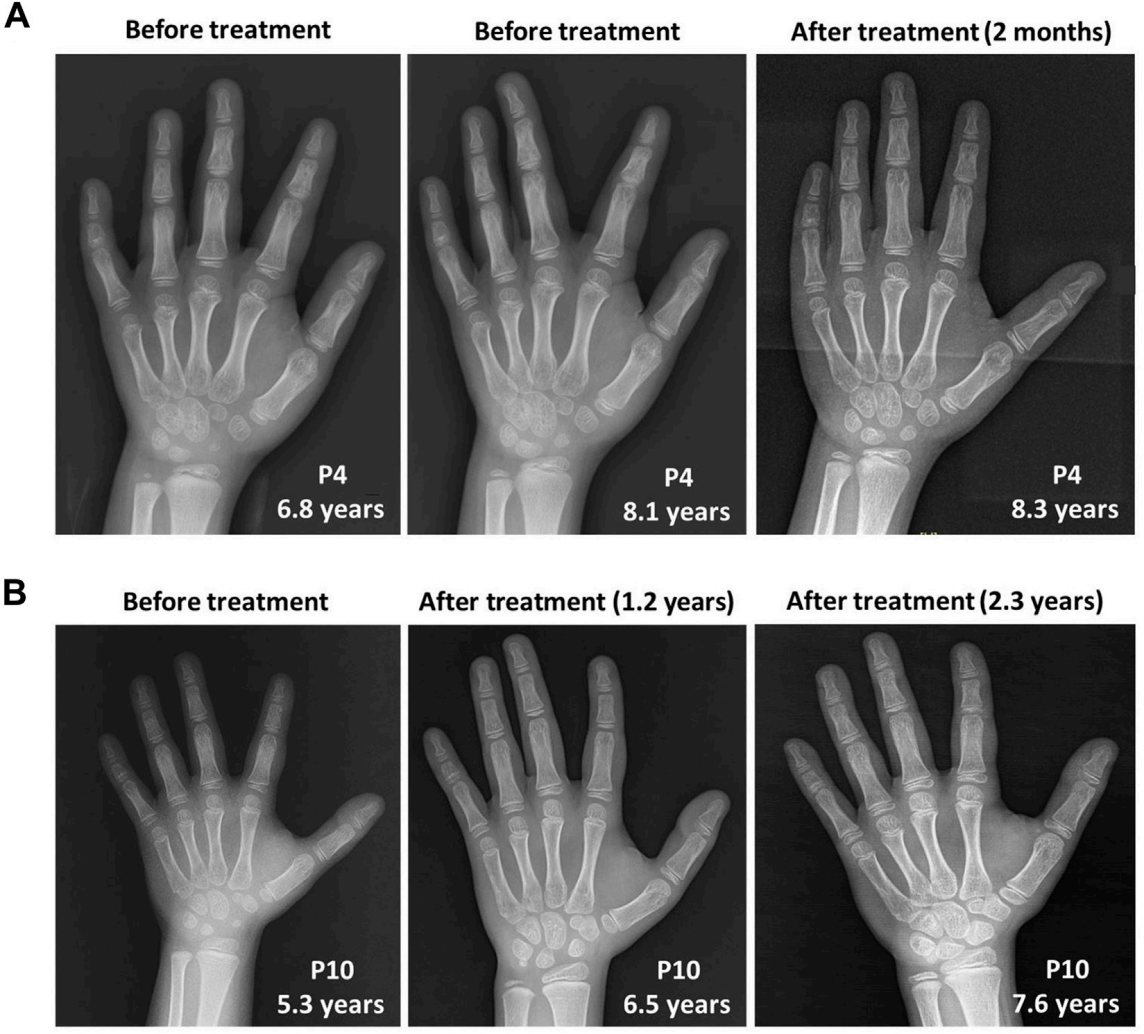
The skeletal X-ray images of two WSS patients before and after rhGH treatment. **(A)** P4. **(B)** P10.

However, growth acceleration of the bone age after rhGH treatment was worth nothing in P10 from approximately 5 years and 9 months at 5.3 years old to 7 years and 10 months at 6.5 years old, and 10 years at 7.6 years old according to the standards of Greulich and Pyle (Greulich and Pyle, 1959). In contrast, the bone age of P4 was consistent with her physical age at the first visit of 6.8 years old, and gradually showed a trend of growth slowdown with a bone age of approximately 7 years and 6 months at 8.1 years old when initiating rhGH treatment. After rhGH treatment for 2 months at 8.3 years old, the bone age of P4 remained the relatively delayed value of 7 years and 6 months (Figure 3).

## 4 Discussion

WSS is a rare disease and far away from well-known in China. To best of our knowledge, 43 Chinese WSS patients have been reported [(Sun et al., 2017; Gao et al., 2018; Li et al., 2018; Chen et al., 2019; Dai et al., 2019; Shangguan et al., 2019; Jinxiu et al., 2020; Wang et al., 2021a; Wang et al., 2021b; Feng et al., 2021; Lee et al., 2021; Luo et al., 2021; Sheppard et al., 2021; Xue et al., 2021; Chen et al., 2022; Wu et al., 2022; Yu et al., 2022), and other 4 literatures in Chinese]. Among them, only one was Chinese cohort study (Li et al., 2018). To better describe clinical and genetic features of WSS patients in China and evaluate therapeutic effects of rhGH, we enrolled this cohort of 11 WSS children.

Consistent with the prior knowledge on WSS, most patients in our cohort manifested typical clinical symptoms of short stature, developmental delay, intellectual disability, facial dysmorphism, and hypertrichosis. However, the frequencies of some features were diverse with three previous studies, the largest WSS cohort, another Chinese WSS cohort and the French WSS cohort (Baer et al., 2018; Li et al., 2018; Sheppard et al., 2021).

As shown in Table 2, short stature was the most common phenotype found in 90.9% children of our cohort, whereas had a lower incidence in other cohorts. The percentage of patients with abnormal corpus callosum was higher in our cohort, whereas strabismus, abnormal dentition, sacral dimple, hypertrichosis elbows, and hypotonia affected fewer patients than other cohorts. Noticeably, aggressive behavior appeared in the other three cohorts with different frequencies, whereas absent in our cohort. Advanced or delayed bone age was revealed in another Chinese WSS cohort (9/10, 90.0%) and the French WSS cohort (12/15, 80.0%) with high frequency, whereas with a lower than 40% frequency in our cohort (2/6, 33.3%) and the largest WSS cohort (11/29, 37.9%). Feeding difficulties were mentioned in more than 60% cases of the largest WSS cohort and the French WSS cohort, whereas approximately 30% in our cohort and another Chinese WSS cohort. The differences in phenotypic frequencies may result from the specificity of ethnicity, limitations of cohort size and whether the symptoms are recognized correctly.

**TABLE 2:** Clinical features of different frequencies comparing with other WSS cohorts.

Clinical features	Our cohort (*n* = 11)	The largest cohort (*n* = 104)	Another Chinese cohort (*n* = 16)	French cohort (*n* = 33)	Chinese patients (*n* = 52)
Short stature (%)	90.9	57.4	75.0	46.9	80.8
Strabismus (%)	9.09	37.5	21.4	21.9	17.6
Abnormal dentition (%)	9.09	57.7	NA	NA	29.4
Sacral dimple (%)	9.09	50.5	25.0	32.0	21.6
Hypertrichosis elbows (%)	36.4	57.0	43.8	61.3	49.0
Hyperactivity (%)	18.2	44.3	12.5	NA	9.8
Aggressive behavior (%)	0.0	33.0	25.0	12.9	7.8
Autism spectrum disorder (%)	9.09	21.3	6.3	6.1	3.9
Hypotonia (%)	45.5	72.4	NA	58.1	21.6
Abnormal corpus callosum (%)	50.0	13.5	NA	13.8	26.9
Feeding difficulties (%)	27.3	66.3	31.3	64.5	23.5
Constipation (%)	18.2	63.8	NA	9.09	3.9
Advanced/delayed bone age (%)	33.3	37.9	90.0	80.0	84.0

NA, not available.

To eliminate the influence arisen from the limited sample size, we further covered 41 previously reported Chinese patients in our analysis. After data incorporation, short stature still was one of the most common phenotypes affecting 80.8% patients. The frequencies of strabismus, abnormal dentition, sacral dimple, hypertrichosis elbows, and aggressive behavior were increased, whereas decreased in hyperactivity, autism spectrum disorder, hypotonia, abnormal corpus callosum, and constipation. Significantly, the frequency of advanced or delayed bone age rose from 33.3% (2/6) in our cohort to 84.0% (21/25) in the set involving 52 Chinese patients (Table 2).

Moreover, our data showed highly phenotypic heterogeneity especially in craniofacial and musculoskeletal system (Supplementary Table S1), suggesting that it is difficult to diagnose WSS based on clinical symptoms alone. Due to the clinical complexity, no diagnostic criteria have been developed for WSS. It is probable that a number of WSS cases are underdiagnosed or misdiagnosed as other syndromes with overlapping phenotypes (Bramswig et al., 2015; Miyake et al., 2016; Baer et al., 2018; Li et al., 2018; Negri et al., 2019; Jinxiu et al., 2020; Demir et al., 2021; Di Fede et al., 2021; Sheppard et al., 2021; Castiglioni et al., 2022; Riedhammer et al., 2022).

In this study, eleven different variants, including three known and eight novel variants, of the *KMT2A* gene were identified in 11 WSS children without a hotspot variant. Of the eight novel variants, seven were point mutations and one was small insertion, resulting in three missense, three nonsense, one splicing, and one frameshift change in the amino acid sequence, which expands the mutational spectrum of this disease.

When mapping to the KMT2A protein, all three missense variants found in this study were located at the CXXC domain, which fits well with the notion that the CXXC domain is a hotspot region for missense variants (Li et al., 2018). Two of the most common phenotypes in our study, short stature and developmental delay, affected all the three patients carrying missense (non-LoF) variants and seven of eight patients carrying LoF variants (87.5%) without a significant difference. However, intellectual disability, another most common phenotype, was manifested in 62.5% patients carrying LoF variants (5/8) and all the three patients carrying missense variants, showing significant difference. Two of three patients carrying missense variants (66.7%) and three of eight patients carrying LoF variants (37.5%) showed hypotonia in our cohort, which differs from the viewpoint that participants with LoF variants are more likely to have hypotonia than those with non-LoF variants (Sheppard et al., 2021). In addition, two of three patients carrying missense variants (66.7%) whereas none of patients carrying LoF variants had seizures, which supports the issue that participants with non-LoF variants are more likely to have seizures (Sheppard et al., 2021).

In WSS patients with GHD, rhGH treatment could achieve satisfactory height gains (Stoyle et al., 2018; Jung et al., 2022). However, two patients (P4 and P10) in our cohort who were not GHD also got benefit from rhGH treatment, suggesting that short stature or failure to thrive not only GHD might be the therapeutic indicator of rhGH in WSS. As sporadic tumors are not present in the germline *KMT2A* variants (Castiglioni et al., 2022), the tumorigenic risk of rhGH treatment in WSS patients is the same as the general population. Furthermore, accelerated skeletal maturation is another problem of rhGH treatment, especially advanced bone age itself is a symptom of WSS (Mendelsohn et al., 2014; Baer et al., 2018; Li et al., 2018; Sheppard et al., 2021). It is difficult to determine the advanced bone age of P10 in our study is caused by rhGH treatment or the disease. In addition, a longer-term follow-up of the bone age is needed in P4, although no growth acceleration of the bone age was revealed in a very short term of 2 months after rhGH treatment. Thus, the role of rhGH treatment in WSS might be double-edged and remains to be further studied.

## 5 Conclusion

Our study adds 11 new patients with WSS, describes their phenotypic and genotypic characteristics, and summarizes the clinical presentations of Chinese WSS patients, which enriches the patient resources and clinical data. Our study also identifies eight novel variants in the *KMT2A* gene, which extends the mutational spectrum. Additionally, our study shares the therapeutic effects of rhGH in two WSS patients without GHD.

## Data Availability

The raw data supporting the conclusions of this article will be made available by the authors, without undue reservation.
