# Supplementary material for: Novel variants and phenotypic heterogeneity in a cohort of 11 Chinese children with Wiedemann-Steiner syndrome
Source: Front Genet. 2023 Mar 21;14:1085210. doi: 10.3389/fgene.2023.1085210 (PMC10070943; doi:10.3389/fgene.2023.1085210)
Supplement: Supplementary file 1 [file Table1.DOC]

**Table S1. Clinical characteristics of Chinese WSS patients**

| **Phenotype** | **Patient** | | | | | | | | | | | **Mean ± SD or Ratio** | | |
| --- | --- | --- | --- | --- | --- | --- | --- | --- | --- | --- | --- | --- | --- | --- |
|  | **P1** | **P2** | **P3** | **P4** | **P5** | **P6** | **P7** | **P8** | **P9** | **P10** | **P11** | **Our cohort** | **Reported patients** | **Total** |
| **Gender** | M | F | M | F | M | M | M | M | M | F | M | 8M/3F | 21M/20F | 29M/23F |
| ***KMT2A* variant** | p.Glu881* | p.Cys1170Phe | p.Arg1811* | p.Gly1168Val | p.Arg1502* | p.Gln3736* | p.Val1347Serfs*24 | p.Cys1155Ser | p.Arg1137* | c.3570-1G>C | ex.2-10 del | / | / | / |
| ***Growth*** |  |  |  |  |  |  |  |  |  |  |  |  |  |  |
| Gestation | Full term | Full term | Full term | Full term | Full term | Full term | Full term | Full term | NA | Full term | Full term | / | / | / |
| Birth length (cm) | 48 | 39 | 46 | 45 | 45 | 50 | NA | 44 | NA | 47 | NA | 45.5 ± 3.3 | 47.6 ± 2.4 | 46.9 ± 2.9 |
| Birth length (SDS) | -1.4 | -6.4 | -2.5 | -2.8 | -3.1 | -0.2 | NA | -3.7 | NA | -1.6 | NA | -2.7 ± 1.9 | -1.4 ± 1.4 | -1.9 ± 1.7 |
| Birth weight (kg) | 2.35 | 1.50 | 2.65 | 2.55 | 2.90 | 2.75 | 3.05 | 2.65 | NA | 2.45 | 3.18 | 2.60 ± 0.47 | 2.84 ± 0.49 | 2.78 ± 0.49 |
| Birth weight (SDS) | -2.7 | -5.7 | -1.8 | -2.0 | -1.1 | -1.5 | -0.7 | -1.8 | NA | -2.3 | -0.4 | -2.0 ± 1.5 | -1.3 ± 1.5 | -1.5 ± 1.5 |
| Prenatal growth retardation | + | + | + | + | + | - | - | + | NA | + | - | 7/10 | 16/35 | 23/45 |
| Age at the first visit (years) | 0.8 | 1.7 | 2.3 | 6.8 | 4.2 | 0.6 | 1.0 | 0.7 | 3.6 | 5.3 | 4.8 | 2.9 ± 2.2 | 5.2 ± 3.3 | 4.5 ± 3.2 |
| Height (SDS) | -4.4 | -6.4 | -3.8 | -3.5 | -4.6 | -1.5 | -3.1 | -3.8 | -3.6 | -2.6 | -2.7 | -3.6 ± 1.3 | -3.4± 1.3 | -3.5 ± 1.3 |
| Weight (SDS) | -6.2 | -6.1 | -3.5 | -2.2 | -4.0 | -4.0 | -3.5 | -4.7 | -2.4 | -1.9 | -2.5 | -3.7 ± 1.5 | -2.2 ± 1.9 | -2.8 ± 1.9 |
| Postnatal growth retardation | + | + | + | + | + | + | + | + | + | + | + | 11/11 | 37/41 | 48/52 |
| Age at the last visit (years) | 1.3 | NA# | NA# | 8.6* | 6.1 | NA# | NA# | 1.1 | NA# | 7.6* | NA# | 3.5 ± 2.8 | 7.5 ± 5.9 | 6.9 ± 5.8 |
| Height (SDS) | -4.1 | NA | NA | -2.5 | -5.0 | NA | NA | -4.5 | NA | -0.3 | NA | -3.3 ± 1.9 | -3.1 ± 1.7 | -3.2 ± 1.7 |
| Weight (SDS) | -4.1 | NA | NA | -2.8 | -3.9 | NA | NA | -3.8 | NA | -0.4 | NA | -3.0 ± 1.5 | -2.2 ± 1.8 | -2.4 ± 1.8 |
| ***Craniofacial features*** |  |  |  |  |  |  |  |  |  |  |  |  |  |  |
| Microcephaly | - | + | - | - | - | - | - | - | - | - | - | 1/11 | 10/40 | 11/51 |
| Cephalus quadratus | - | - | - | + | - | - | - | - | - | - | - | 1/11 | 0/40 | 1/51 |
| Prominent forehead | + | - | - | - | - | - | - | - | + | - | - | 2/11 | 9/40 | 11/51 |
| Synophrys | - | - | - | - | - | - | - | - | - | + | - | 1/11 | 8/40 | 9/51 |
| Eyebrow lateral flare | - | - | + | - | - | - | - | - | + | - | + | 3/11 | 0/40 | 3/51 |
| Arched eyebrows | - | - | - | - | - | - | - | - | + | + | - | 2/11 | 16/40 | 18/51 |
| Small palpebral fissures | - | - | + | - | - | - | - | - | - | + | + | 3/11 | 8/40 | 11/51 |
| Downslanted palpebral fissures | - | - | + | + | - | - | - | - | + | - | - | 3/11 | 17/40 | 20/51 |
| Hypertelorism | - | + | + | - | + | - | - | - | - | - | - | 3/11 | 26/40 | 29/51 |
| Small ears | - | - | + | - | - | - | - | - | - | - | - | 1/11 | 0/40 | 1/51 |
| Prominent ears | - | - | - | - | + | - | - | - | - | - | - | 1/11 | 0/40 | 1/51 |
| Low-set ears | + | - | + | + | + | - | - | + | + | - | - | 6/11 | 17/40 | 23/51 |
| Auricular anomaly | + | - | - | - | - | - | - | - | - | - | - | 1/11 | 3/40 | 4/51 |
| Flat nasal bridge | + | - | + | - | - | - | - | + | - | + | + | 5/11 | 27/40 | 32/51 |
| Short columella | - | - | + | + | - | - | - | - | + | - | - | 3/11 | 0/40 | 3/51 |
| Broad nasal tip | - | - | - | + | - | - | - | - | - | + | - | 2/11 | 8/40 | 10/51 |
| Anteverted nares | - | + | + | + | - | - | - | - | + | - | - | 4/11 | 0/40 | 4/51 |
| Thin upper lip | + | - | - | + | - | - | - | - | - | - | + | 3/11 | 15/40 | 18/51 |
| High-arched palate | + | - | - | - | - | - | - | + | - | + | - | 3/11 | 20/40 | 23/51 |
| Long philtrum | + | - | - | - | - | - | - | - | - | - | - | 1/11 | 19/40 | 20/51 |
| Downturned corners of the mouth | - | - | + | - | - | - | - | - | - | - | - | 1/11 | 17/40 | 18/51 |
| Micrognathia | - | - | - | - | - | - | - | + | - | - | - | 1/11 | 7/40 | 8/51 |
| Retrognathia | - | - | - | - | + | - | - | - | - | - | - | 1/11 | 0/40 | 1/51 |
| Webbed neck | - | - | - | - | + | - | - | - | - | - | - | 1/11 | 0/40 | 1/51 |
| ***Ocular abnormalities*** |  |  |  |  |  |  |  |  |  |  |  |  |  |  |
| Strabismus | - | + | - | - | - | - | - | - | - | - | - | 1/11 | 8/40 | 9/51 |
| Astigmatism | - | - | + | - | - | - | - | - | - | - | - | 1/11 | 1/40 | 2/51 |
| ***Auditory issues*** |  |  |  |  |  |  |  |  |  |  |  |  |  |  |
| Hearing impairment | - | + | - | - | - | + | - | - | - | - | - | 2/11 | 0/40 | 2/51 |
| ***Musculoskeletal problems*** |  |  |  |  |  |  |  |  |  |  |  |  |  |  |
| Short stature | + | + | + | + | + | - | + | + | + | + | + | 10/11 | 32/41 | 42/52 |
| Slim and muscular build | + | + | - | - | - | + | + | + | - | - | + | 6/11 | 4/40 | 10/51 |
| Abnormal dentition | - | - | - | - | - | - | - | - | - | + | - | 1/11 | 14/40 | 15/51 |
| Spinal anomaly | - | - | - | - | + | - | - | - | - | - | - | 1/11 | 1/40 | 2/51 |
| Sacral cleft | - | - | + | - | - | - | - | - | - | - | - | 1/11 | 0/40 | 1/51 |
| Sacral dimple | - | - | + | - | - | - | - | - | - | - | - | 1/11 | 10/40 | 11/51 |
| Rib eversion | - | - | - | + | - | - | - | - | - | - | - | 1/11 | 1/40 | 2/51 |
| Absence of the 12th rib | - | - | - | - | + | - | - | - | - | - | - | 1/11 | 0/40 | 1/51 |
| Lower limb deformity | - | - | - | + | - | - | - | - | - | - | - | 1/11 | 0/40 | 1/51 |
| Broad first digits | - | - | - | - | - | - | - | - | - | - | + | 1/11 | 0/40 | 1/51 |
| Toe deformity | - | - | - | - | - | - | - | + | - | - | - | 1/11 | 11/40 | 12/51 |
| Transverse palmar creases | - | + | - | - | - | - | - | - | - | - | - | 1/11 | 0/40 | 1/51 |
| Advanced bone age | NA | NA | + | - | - | NA | NA | NA | - | - | - | 1/6 | 3/19 | 4/25 |
| Delayed bone age | NA | NA | - | - | + | NA | NA | NA | - | - | - | 1/6 | 15/19 | 17/25 |
| ***Hairness*** |  |  |  |  |  |  |  |  |  |  |  |  |  |  |
| Thick hairs | - | - | - | - | - | - | - | + | + | + | + | 4/11 | 26/40 | 30/51 |
| Thick eyebrows | - | - | - | + | + | - | - | + | + | + | + | 6/11 | 19/40 | 25/51 |
| Long eyelashes | + | + | - | + | + | - | - | - | - | + | + | 6/11 | 26/40 | 32/51 |
| Hypertrichosis cubiti | + | - | - | - | + | - | - | - | - | + | + | 4/11 | 21/40 | 25/51 |
| Hypertrichosis of the back | + | + | - | + | + | - | - | + | - | + | + | 7/11 | 29/40 | 36/51 |
| Hypertrichosis of lower limbs | + | - | - | - | + | - | - | + | - | + | + | 5/11 | 23/40 | 28/51 |
| Low hairline | - | - | - | - | + | - | - | - | - | + | - | 2/11 | 24/40 | 26/51 |
| ***Neurological symptoms*** |  |  |  |  |  |  |  |  |  |  |  |  |  |  |
| Developmental delay | + | + | + | + | - | + | + | + | + | + | + | 10/11 | 34/41 | 44/52 |
| Intellectual disability | + | + | + | + | - | + | + | + | - | - | + | 8/11 | 36/41 | 44/52 |
| Hyperactivity | - | - | - | + | - | - | - | - | - | + | - | 2/11 | 3/40 | 5/51 |
| Aggressive behavior | - | - | - | - | - | - | - | - | - | - | - | 0/11 | 4/40 | 4/51 |
| Autism spectrum disorder | - | - | - | + | - | - | - | - | - | - | - | 1/11 | 1/40 | 2/51 |
| Hypotonia | + | - | - | + | - | + | + | + | - | - | - | 5/11 | 6/40 | 11/51 |
| Seizure | - | - | - | - | - | - | - | + | - | - | - | 1/11 | 1/40 | 2/51 |
| Abnormal EEG | - | NA | - | + | NA | NA | NA | - | NA | NA | NA | 1/4 | 1/3 | 2/7 |
| Abnormal corpus callosum | + | + | - | - | + | + | - | + | NA | - | - | 5/10 | 2/16 | 7/26 |
| Delayed myelination | + | + | - | + | - | - | - | - | NA | - | - | 3/10 | 2/16 | 5/26 |
| Small pituitary | - | - | - | - | + | - | - | - | NA | - | - | 1/10 | 0/16 | 1/26 |
| ***Gastrointestinal phenotypes*** |  |  |  |  |  |  |  |  |  |  |  |  |  |  |
| Constipation | - | - | - | + | - | - | - | + | - | - | - | 2/11 | 0/40 | 2/51 |
| Feeding difficulties | - | + | - | - | + | - | - | + | - | - | - | 3/11 | 9/40 | 12/51 |
| ***Cardiovascular defects*** |  |  |  |  |  |  |  |  |  |  |  |  |  |  |
| Patent ductus arteriosus | + | + | - | - | NA | NA | NA | + | - | NA | + | 4/7 | 4/29 | 8/36 |
| Patent foramen ovale | + | - | + | - | NA | NA | NA | + | - | NA | - | 3/7 | 3/29 | 6/36 |
| Atrial septal defect | - | - | - | - | NA | NA | NA | + | - | NA | - | 1/7 | 1/29 | 2/36 |
| Pulmonary arterial hypertension | - | - | - | - | NA | NA | NA | + | - | NA | - | 1/7 | 0/29 | 1/36 |
| Arterio-arterial fistula | - | - | - | + | NA | NA | NA | - | - | NA | - | 1/7 | 0/29 | 1/36 |
| Abnormal ECG | - | NA | - | + | - | NA | NA | + | NA | - | + | 3/7 | 1/9 | 4/16 |
| ***Urogenital*** ***abnormalities*** |  |  |  |  |  |  |  |  |  |  |  |  |  |  |
| Renal malformation | NA | NA | + | - | + | NA | NA | NA | NA | - | - | 2/5 | 1/7 | 3/12 |
| Ureterocele | NA | NA | - | - | + | NA | NA | NA | NA | - | - | 1/5 | 0/7 | 1/12 |
| Bladder diverticula | NA | NA | - | - | + | NA | NA | NA | NA | - | - | 1/5 | 0/7 | 1/12 |
| Urinary tract infections | - | - | - | + | - | - | - | - | - | - | - | 1/11 | 0/40 | 1/51 |
| Urinary frequency | - | - | + | + | - | - | - | - | - | - | - | 2/11 | 0/40 | 2/51 |
| Cryptorchidism | - | IA | - | IA | - | - | - | + | + | IA | - | 2/8 | 4/20 | 6/28 |
| ***Immunological dysfunction*** |  |  |  |  |  |  |  |  |  |  |  |  |  |  |
| Recurrent infections | - | + | - | + | + | - | - | + | - | - | - | 4/11 | 2/40 | 6/51 |
| History of eczema | - | - | + | + | + | - | - | + | - | - | - | 4/11 | 0/40 | 4/51 |
| History of allergy | - | - | + | + | + | - | - | + | - | - | - | 4/11 | 0/40 | 4/51 |

# These patients were lost to follow-up; * After treatment with rhGH.

SDS, standard deviation score; SD, standard deviation; M, male; F, female; NA, not available; IA, inapplicable.
